# Detecting microcephaly and macrocephaly from ultrasound images using artificial intelligence

**DOI:** 10.1186/s12880-025-01709-x

**Published:** 2025-05-26

**Authors:** Abraham Keffale Mengistu, Bayou Tilahun Assaye, Addisu Baye Flatie, Zewdie Mossie

**Affiliations:** 1 Department of Health Informatics, College of Medicine Health Science, Debre Markos University, Debre Markos, Ethiopia; 2 Department of Information Technology, Institute of Technology, Debre Markos University, Debre Markos, Ethiopia

**Keywords:** Microcephaly, Macrocephaly, Congenital abnormality, HC, BPD

## Abstract

**Background:**

Microcephaly and macrocephaly, which are abnormal congenital markers, are associated with developmental and neurologic deficits. Hence, there is a medically imperative need to conduct ultrasound imaging early on. However, resource-limited countries such as Ethiopia are confronted with inadequacies such that access to trained personnel and diagnostic machines inhibits the exact and continuous diagnosis from being met.

**Objective:**

This study aims to develop a fetal head abnormality detection model from ultrasound images via deep learning.

**Methods:**

Data were collected from three Ethiopian healthcare facilities to increase model generalizability. The recruitment period for this study started on November 9, 2024, and ended on November 30, 2024. Several preprocessing techniques have been performed, such as augmentation, noise reduction, and normalization. SegNet, UNet, FCN, MobileNetV2, and EfficientNet-B0 were applied to segment and measure fetal head structures using ultrasound images. The measurements were classified as microcephaly, macrocephaly, or normal using WHO guidelines for gestational age, and then the model performance was compared with that of existing industry experts. The metrics used for evaluation included accuracy, precision, recall, the F1 score, and the Dice coefficient.

**Results:**

This study was able to demonstrate the feasibility of using SegNet for automatic segmentation, measurement of abnormalities of the fetal head, and classification of macrocephaly and microcephaly, with an accuracy of 98% and a Dice coefficient of 0.97. Compared with industry experts, the model achieved accuracies of 92.5% and 91.2% for the BPD and HC measurements, respectively.

**Conclusion:**

Deep learning models can enhance prenatal diagnosis workflows, especially in resource-constrained settings. Future work needs to be done on optimizing model performance, trying complex models, and expanding datasets to improve generalizability. If these technologies are adopted, they can be used in prenatal care delivery.

**Clinical trial number:**

Not applicable.

## Background

Fetal head anomalies are differences in growth rates, structure, or outline shape that may differ from normal when the development of the fetal head is considered [1, 2]. Conditions that might estimate possible health complications and incidences related to developmental malformations include congenital genetic anomalies, infections, or other agents affecting normal fetal development conditions, such as certain maternal infections or toxins during pregnancy [3–5].

Microcephaly and macrocephaly are two congenital conditions characterized by head sizes that are either abnormally small or abnormally large [6, 7]. These conditions are known to result in severe neurological and developmental issues [8]. Timely detection of abnormalities via ultrasound is essential for appropriate medical intervention. Recently, there has been high interest in the identification of microcephaly around the world following its return into the limelight during the outbreak of the Zika virus, when many cases were reported [8]. While macrocephaly may be less common, this does not mean that its severity, as a condition affecting the development of the fetus, cannot be underestimated. The early detection of such conditions allows for the planning of appropriate postnatal care, if needed, for the best outcomes for the infants affected [4, 9].

Microcephaly is a condition characterized by the fetal head usually being too small, and therefore accompanied by both cognitive and physical disabilities [10], whereas macrocephaly involves having an abnormally large head [8], which is usually associated with brain defects or alterations. The detection of such abnormalities by deep learning, especially through CNNs, has been identified as one of the ways of resolving issues associated with manual methods of detection [8, 12]. Ultrasound images can be processed efficiently for detecting fetal head abnormalities and providing real feedback almost instantly, hence making the technology very useful in areas where skilled health workers are not readily available [13, 14].

There is advanced prenatal health care in resource-rich countries, employing ultrasound imaging through a sonographer to monitor fetal development [12, 15, 16]. Specialized prenatal health care, like this, exists very little in most disadvantaged parts of the world. Therefore, the only way to measure these two key parameters is manual measurement via ultrasound, which is very slow and operator-dependent to the extent that some cases may go unnoticed, especially in far remote regions that are poorly served, where microcephaly and macrocephaly are more common [15].

The motivation for this study is the need to reduce the gap in prenatal diagnostic care between well-resourced and underprivileged regions. Therefore, the integration of CNN-based models for fetal head measurements could be a scaling solution to the problems of operator dependency challenges and variability in image quality. The current study not only fills a significant gap in healthcare but also provides one possible way forward regarding the use of AI for medical diagnostics to increase the global accessibility and reliability of prenatal care. To maximize detection and ensure minimal human error during operator-dependent activities, automated detection of fetal head abnormalities is necessary for appropriate patient care. Therefore, this study aims to fill this gap by developing an automated model for anomaly detection in a fetus’s head via deep learning models.

This study aimed to devise a deep learning-based model for the automatic detection of microcephaly and macrocephaly from fetal ultrasound images and further assess its effectiveness. The importance of this study cannot be overemphasized, given that this will dramatically improve diagnostic accuracy and increase access to prenatal care for so many underserved parts of the world where traditional methods are sorely lacking. Automating the diagnosis could increase the uniformity with which fetal head anomalies are diagnosed within the timeframe, while lessening the workload for healthcare workers and improving the health outcomes of babies with other developmental issues.

## Related works

### Summary of related works

Recent studies have employed diverse methodologies to advance fetal ultrasound segmentation, though persistent challenges remain (Table 1). Approaches include U-Net fine-tuning hybrid attention mechanisms, and uncertainty-aware framework, which aim to improve accuracy in complex scenarios. However, common limitations across these works include limited data availability, variability in ultrasound image quality and anatomical complexities such as boundary ambiguity or fetal pose variability. Model-specific issues, such as suboptimal fine-tuning strategies and imperfect uncertainty modeling, further hinder generalizability. These challenges underscore the need for robust datasets, enhanced image preprocessing, and adaptive algorithms to address the heterogeneity of clinical ultrasound data and fetal developmental stages.

**Table 1:** Related works and their limitations

No.	Reference (Author and Year)	Methodology	Limitations
1	Wang et al., 2024	Fine-tuning U-Net	Limited data availability in low-resource settings; challenges in achieving optimal fine-tuning strategies.
2	Yang et al., 2020	Hybrid attention mechanism	Challenges include poor image quality, boundary ambiguity, and appearance variability across different fetal poses and gestational ages.
3	Nagabotu et al., 2024	Improved U-Net model	Challenges include noisy ultrasound images and variability in fetal head development; overlapping sutures and blurred boundaries.
4	Chen et al., 2021	Deep learning-based Segmentation	Model performance is influenced by the quality and variability of ultrasound images across different trimesters.
5	Sobhaninia et al., 2019	Multitask deep CNN	Need for large, annotated datasets; challenges posed by variability in ultrasound image quality.
6	Thaler et al., 2022	Optimized deep learning	Relatively small dataset; potential variability in image quality affecting generalizability.
7	Lei et al., 2024	Uncertainty-aware automated measurement	Data uncertainty impacts measurement reliability; it requires refinement in uncertainty modeling techniques.

## Methods and materials

### Study area and period

The study was conducted across three healthcare facilities in Ethiopia, including hospitals and clinics. The recruitment period for this study started on November 9, 2024, and ended on November 30, 2024. This study gathered various ultrasound images to represent different healthcare settings. The selected sites for this study included Dessie Comprehensive Specialized Hospital, Dejen Primary Hospital, and the Family Guidance Association Debre Markos Clinic. Such places were chosen so that both high-tech and low-tech contexts may be used for a comprehensive understanding of the model’s performance in different situations. Model experimentation and evaluation, including report writing, were performed from December 1 to January 30, 2025.

### Study design

This study identified the best segmentation model via a deep learning approach. Additionally, a rule-based system is developed that uses the logic of percentile ranges for HCs and BPD patients to classify the outcomes as described by the WHO. The study uses images collected at a time with a collaborative campaign; it has a cross-sectional study design.

### Data sources

#### Description of the dataset

Ultrasound images of the heads of fetuses annotated with the measurements of HCs and BPD patients were considered. Data were collected from Dessie Comprehensive Specialized Hospital, Dejen Primary Hospital, and the Family Guidance Association Debre Markos Clinic (Table 2). Images were captured from both the second and third trimesters proportionally.

**Table 2:** Datasets collected by facility and trimester

No.	Facility Name	Images collected	
		2nd trimester	3rd trimester
1	Dessie Hospital	200	200
2	Dejen Hospital	100	100
3	FGAE Clinic	50	50

### Data collection

These ultrasound images of a normally shaped head with no visible deformed part have been collected in collaboration with healthcare institutes that target pregnant women for routine prenatal examinations. Overall, 700 images have been gathered in the hope of having a broad variety of head sizes and states of fetuses. The total number is chosen as a balance between capacity and modeling precision. Data balancing was maintained in terms of the trimester; an equal amount of data was collected for each trimester. From these images, the values of HCs and BPD patients were measured in 200 patients for later comparison.

The targeted population consists of pregnant women attending routine ultrasound studies in their second or third trimesters at collaborating hospitals or clinics. Informed consent for the collection of data has been obtained at the policy or administrative level within the institutions involved in participating hospitals/clinics, since obtaining such permission through individual signing has become unwieldy. More fundamentally, although these images contain no personal identifiers/demographics from participating patients, the information taken down would infringe on policies laid down in a research context of ethics on breaches.

**Fig. 1:**
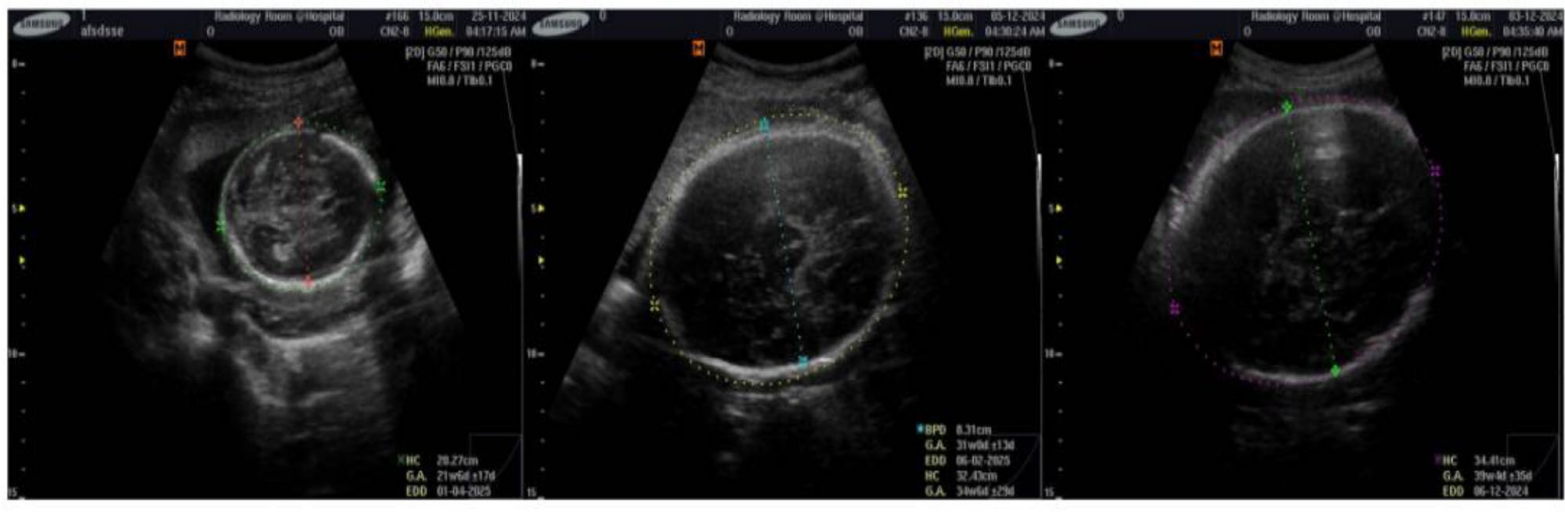
Raw images annotated and measured by gynecologists

Data collection was performed according to standardized protocols by qualified sonographers or gynecologists at healthcare institutions to maintain the quality of the images (Fig. 1).

All the ultrasound images annotated by gynecologists are considered the critical benchmark in the validation of model performance for predicting fetal head boundaries. These annotated images include those of HCs and BPD patients, which are two important clinical measures indicating fetal growth and abnormalities such as microcephaly or macrocephaly. These facts ensure that clinical measurement segmentation predictions by the model are quantitatively comparable since manual annotations by the gynecologist provide ground truth data of high reliability.

### Image preprocessing

First, images collected from different facilities had to be organized consistently before model input during operation. Several techniques of image preprocessing have been employed to prepare the dataset in this research for model training to further improve the performance. This involves various augmentations, including data augmentation, and horizontal and vertical flipping. These augmentations are performed via random horizontal and vertical flipping with a probability of 50% to obtain a good variety of images in the dataset to prevent overfitting. Resizing: All the images and masks are brought to the same dimension, height = 128 pixels and width = 192 pixels, which is accomplished via the Resize transformation in the albumentation library. For binary mask processing, refinement of the annotation masks was performed such that the segmented regions were filled to produce accurate binary segmentation maps and reduce noise. In addition, the normalization and conversion steps included converting images and masks into tensors. This transformation ensures compatibility with PyTorch models and scales image pixel values to the range [0, 1]. Finally, visualization functions were used to cross-check the correctness of the preprocessing steps by showing images, masks, and boundaries with boundaries highlighted. In this way, these preprocessing techniques collectively ensure a robust pipeline for training segmentation models. All the images are annotated for training along with a gynecologist via the VGG 19 image annotator software.

### Operational definitions

#### Microcephaly

An HC that is smaller than the standard deviation for gestational age.

#### Macrocephaly

An HC that exceeds the normal range for gestational age.

### Modeling framework

We follow a semi-supervised learning approach to develop a robust modeling framework for fetal ultrasound image segmentation, wherein the labeled data are utilized together with unlabeled data. Such a strategy is indeed very efficient in medical imaging, where it is usually difficult to acquire large labeled datasets because of expert annotations. The proposed framework uses unlabeled ultrasound images along with a small amount of labeled data for better generalization and segmentation of the model.

Central to our framework is the integration of CNN-based architectures for image segmentation and classification tasks. CNNs have shown outstanding performance in capturing spatial hierarchies and patterns within medical images, making them very suitable for delineating complex anatomical structures in ultrasound scans. In our approach, the CNN is trained to segment the key fetal biometric parameters HC and BPD, which are critical for assessing fetal development.

**Fig. 2:**
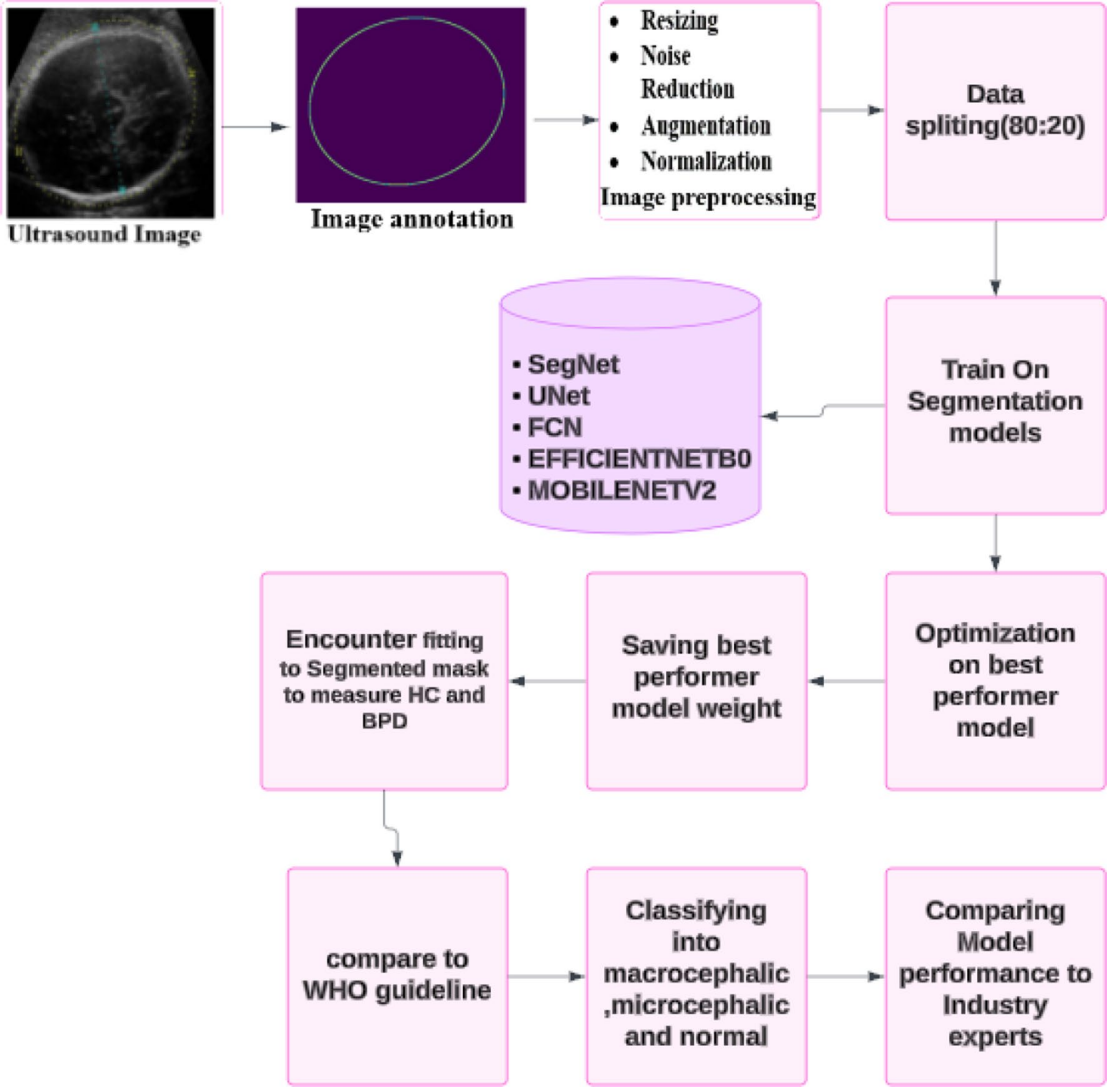
Model framework

We used standard deviation measurements of HCs and BPD patients at specific gestational ages (GAs) to establish a benchmark of normal fetal growth, using standards defined by the WHO. These reference ranges are important in the clinical identification of abnormal growth. We align our model outputs to ensure clinically relevant segmentations that can help in the early detection of potential anomalies (Fig. 2).

### Models

The selection of appropriate models and architectures is very important for the accurate and efficient analysis of fetal ultrasound images. Several deep-learning architectures were explored in this work to address the challenges of segmentation and classification in medical imaging. The proposed models are SegNet, UNet, fully convolutional networks, EfficientNet-B0, and MobileNetV2. These models represent a spectrum of architectural diversity and efficiency [17–19]. Each of these architectures provides unique strengths to the task, offering a balance between accuracy, computational efficiency, and suitability for ultrasound imaging. This work tends to leverage the power of such diverse architectures toward the development of a robust framework in the analysis of fetal ultrasound images. Each model has advantages, but the final choice will depend on task requirements, available computational resources, and the need for scalability in clinical settings.

### Optimized SegNet model architecture

**Fig. 3:**
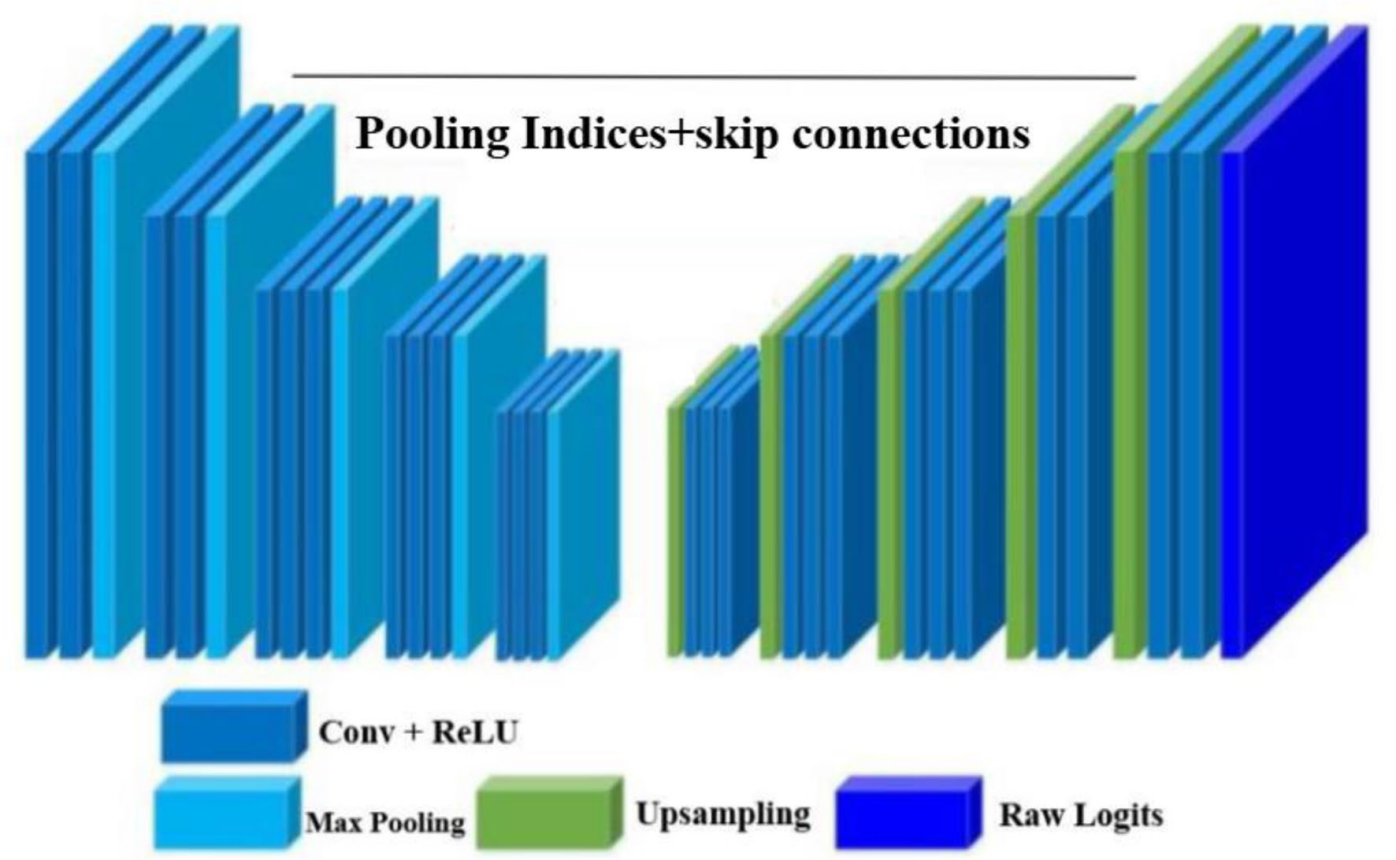
The optimized SegNet model architecture used for the final training

In this work, the SegNet architecture implemented here is specifically designed for pixel-wise image segmentation and performs especially well on medical imaging tasks. It consists of a strong encoder-decoder framework that effectively captures spatial features and reconstructs high-resolution segmentation maps. This model starts with an input layer that takes 128 × 192 × 3 images, where the three channels correspond to the RGB color space. This layer directly feeds the data into the encoder without any additional transformations or activations.

The encoder is structured into five hierarchical blocks of convolutional layers, followed by max pooling to reduce the spatial dimensions while extracting features at a progressively higher level. First, the encoder block consists of two convolutional layers with a filter size of 3 × 3, a stride of 1, and an output channel of 64, followed by max pooling, which reduces the spatial dimensions to 64 × 96 × 64. Similarly, the subsequent encoder blocks progressively increase the number of filters while reducing the spatial dimensions by half until the fifth block ends with feature maps of size 4 × 6 × 512 after three convolutional layers, followed by max pooling (Fig. 3).

The decoder mirrors the encoder structure and uses up-sampling layers to restore the feature maps spatially. Furthermore, each up-sampling step is followed by convolutional layers that process the up-sampled feature maps. For example, in the fifth decoder block, up-sampling increases the dimensions to 8 × 12 × 512, and three convolutional layers further process these features. This structure symmetrically continues until the last decoder block has restored the dimensions to the input dimensions of 128 × 192 × 64 (Table 3).

The model finally concludes with a pure output layer of convolution with a kernel of size 1 × 1 to match the refined feature maps to the required output channels, K, corresponding to segmentation classes, K = 1 for binary and 128 × 192 × 1 dimension in the case of segmentation probabilities at the pixel level. After every convolutional layer, both in the encoder and decoder, ReLU activation is used to introduce nonlinearity; this helps the model learn complex patterns effectively. In addition, sigmoid activation on the output is applied during evaluation, which transforms the logits into probabilities for binary segmentation.

This implementation involves many refinements in the standard SegNet architecture: a parameterized design for flexibility, bilinear up-sampling to simplify the decoder, and additional convolutional layers for refined feature extraction. These enhancements ensure the robustness of the model, its computational efficiency, and adaptability to the specific challenges presented by medical image segmentation, making it particularly suitable for tasks such as fetal head segmentation.

**Table 3:** Selected model SegNet architecture

Layer	Type	Output feature map dimensions	Filter size	Stride
Input	Input Layer	128 × 192 × 3	-	-
Encoder 1	Convolution (x2)	128 × 192 × 64	3 × 3	1
	Max Pooling	64 × 96 × 64	2 × 2	2
Encoder 2	Convolution (x2)	64 × 96 × 128	3 × 3	1
	Max Pooling	32 × 48 × 128	2 × 2	2
Encoder 3	Convolution (x3)	32 × 48 × 256	3 × 3	1
	Max Pooling	16 × 24 × 256	2 × 2	2
Encoder 4	Convolution (x3)	16 × 24 × 512	3 × 3	1
	Max Pooling	8 × 12 × 512	2 × 2	2
Encoder 5	Convolution (x3)	8 × 12 × 512	3 × 3	1
	Max Pooling	4 × 6 × 512	2 × 2	2
Decoder 5	Upsampling	8 × 12 × 512	-	-
	Convolution (x3)	8 × 12 × 512	3 × 3	1
Decoder 4	Upsampling	16 × 24 × 512	-	-
	Convolution (x3)	16 × 24 × 512	3 × 3	1
Decoder 3	Upsampling	32 × 48 × 256	-	-
	Convolution (x3)	32 × 48 × 256	3 × 3	1
Decoder 2	Upsampling	64 × 96 × 128	-	-
	Convolution (x2)	64 × 96 × 128	3 × 3	1
Decoder 1	Upsampling	128 × 192 × 64	-	-
	Convolution (x2)	128 × 192 × 64	3 × 3	1
Output	Convolution	128 × 192×K	1 × 1	1

### Mathematical modeling optimized SegNet model architecture

#### Encoder-Decoder architecture

It is designed for pixel-wise segmentation, combining convolutional layers for feature extraction (encoder) and up-sampling layers for mask reconstruction (decoder).

### Encoder (Feature extraction)

- **Convolutional Layer**:
  Each encoder block applies a convolution operation:

$$\:{\varvec{Z}}^{\left(l\right)}\hspace{0.17em}=\hspace{0.17em}{\varvec{W}}^{\left(l\right)}*{\varvec{A}}^{(l-1)}\hspace{0.17em}+\hspace{0.17em}{\varvec{b}}^{\left(l\right)}$$

where W^(*l*)^ = learnable weights, **A**^(*l*−1)^ = input activations, **b**^(*l*)^ = biases, and ∗ = convolution operator.

- **Batch Normalization**:
  Normalizes activations to stabilize training:

$$\:{\hat {\textbf{Z}}^{\left( l \right)}} = \gamma \:\frac{{{{\textbf{Z}}^{\left( l \right)}} - \mu \:}}{{\sqrt {\sigma {\:^2} + \in } }} + \beta \:$$

where µ, σ^2^ = mean/variance of Z^(l)^, γ, *β* = learnable parameters, and $\in$ = small constant.

- **ReLU Activation**:
  Introduces non-linearity:

$$\:{A^{\left( l \right)}} = max\:(0,\:{\hat Z^{\left( l \right)}})$$

- **Max Pooling**:
  Reduces spatial dimensions while preserving critical features. Pooling indices (locations of max values) are stored for decoder up-sampling.

### Decoder (Mask reconstruction)

- **Up-sampling with Pooling Indices**:
  Uses saved indices from the encoder to spatially relocate features, preserving boundary details.

$$\:{A}_{up}^{\left(l\right)}=Upsample\:({A}^{\left(l\right)},\:Poo{l}_{indices})$$

- **Convolutional Layers**:
  Refines up-sampled features to reconstruct the segmentation mask.

### Loss function

A hybrid loss combining **Binary Cross-Entropy (BCE)** and **Dice Loss** optimizes both pixel-wise accuracy and mask overlap:

- Binary Cross-Entropy (BCE):

$${\mathcal{L}_{BCE}} = - \frac{1}{N}\sum {\:_{i = 1}^N} [{y_i}log\left( {{p_i}} \right) + \left( {1 - {y_i}} \right)log\left( {1 - {p_i}} \right)]$$

Where *yi* = ground truth, *pi* = predicted probability, *N* = total pixels.

- **Dice Loss**: Maximizes overlap between predicted (*P*) and ground truth (*G*) masks:

$${\mathcal{L}_{Dice}} = 1 - \frac{{2\left| {P \cap G} \right|}}{{\left| P \right| + \left| G \right|}}$$

- **Total loss**

$${\mathcal{L}_{total}} = \alpha \:{\mathcal{L}_{BCE}} + \left( {1 - \alpha \:} \right){\mathcal{L}_{Dice}}$$

We set *α* = 0.5 to balance both terms.

The architecture’s mathematical foundation, combining hierarchical feature extraction, spatial up-sampling, and hybrid loss optimization, underpins its success in automating fetal head biometrics. We appreciate the opportunity to elaborate on these details and will incorporate this section into the revised manuscript.

## Model training

### Software and hardware requirements

In this research, the computational environment used an Intel(R) Core (TM) i7-8650U CPU running at 1.90 GHz with 16 GB of installed RAM. The hardware configuration has been used in training and performing deep-learning models related to the segmentation and classification of fetal ultrasound images. High-resolution computation with ultrasound images was easily managed with the architecture of the 64-bit operating system.

On the software side, Python was used as the primary programming language, with most of its deep learning libraries and frameworks. Specifically, PyTorch was utilized for the creation and training of the CNN models proposed in this study, which allows the efficient use of complicated neural network architectures. For image preprocessing and augmentation purposes, OpenCV and Albumentations were used in this study. OpenCV involves basic image processing, and Albumentation provides a fast, flexible image augmentation library. This improved our model’s robustness and further generalized its performance. This combination of hardware and software resources provided us with a firm and efficient base for our experiments, ensuring that the computational demands needed for deep learning-based fetal ultrasound image analysis would be well supported.

### The training processes

This training of the SegNet model has been performed in the same controlled environment as the other four models, namely, UNet, FCN, MobileNetV2, and EfficientNet-B0. The dataset used is split into 80% for training and 20% for testing to ensure that there is a strong generalization of the models being measured. The performances were further fine-tuned for both the U-Net and SegNet models, each trained at three different epoch levels: 25, 50, 100, and 125. This allowed us to analyze how the performance of the models changes with training time.

The iterative update of model weights was performed during training, and the performance metrics were continuously evaluated on both the training and validation datasets after each epoch. Different regularization techniques, including dropout, have been used to reduce overfitting issues and enhance the ability of models to generalize to unseen data. In addition, checkpoints were used to save the states of the model from time to time to recover or experiment with other configurations.

### Model optimization

The optimization is usually performed by delicately choosing a combination of an appropriate loss function, optimizer, and learning rate schedule, along with training strategies for the segmentation model.

It leverages performance by using binary cross-entropy loss along with Dice loss. The BCE loss affects the probabilities that were predicted and the actual ground truth values, considering every pixel, as it performs binary classification on them. Moreover, Dice loss has been incorporated for direct optimization of the Dice coefficient, one of the very common metrics of segmentation, which describes the overlap between the predicted and ground truth masks. This combined loss function ensures both accurate pixel-level classification and overall mask quality.

The Adam optimizer was used with a learning rate of 0.001 to adapt the learning rates for each parameter. Adam is popularly known to be very effective in improving convergence speed and stability while training. Finally, a ReduceLROnPlateau scheduler was implemented to perform dynamic learning rate adjustments. When the validation loss has stopped improving, that is, plateaus for a certain number of epochs (patience = 20), the learning rate is reduced by a factor of 0.5. This adjustment fine-tunes the training process, especially when progress slows, enhancing the model’s ability to reach an optimal solution. The training loop processes data in mini-batches, thus enabling efficient computation. Gradients are calculated through backpropagation, and weights are updated via the Adam optimizer (Table 4).

The model also saves weights at every best validation loss so that the best performance is locked in. Sanity checks at the beginning of training are also implemented to verify whether the data pipeline and model work as expected. The Dice coefficient is computed in batches concerning segmentation performance. This metric provides a direct measure of overlap between the predicted masks and ground truth, complementing the loss functions. While implicit, early stopping behavior within the design of a learning rate scheduler ensures that once there are no improving validation losses, there is no major update from the model to avoid overfitting or training the model unnecessarily. The generated segmentation masks are highly scaled in space, and the up-sampling process is involved during the decoding phase in building a high-scale segmentation mask.

**Table 4:** Key components and parameters used in optimizing our segmentation model

Component	Parameter	Value
Loss Function	Binary Cross-Entropy (BCE)	Applied
	Dice Loss	Applied
Optimizer	Type	Adam
	Learning Rate	0.001
Learning Rate Scheduler	Type	ReduceLROnPlateau
	Mode	‘min’
	Factor	0.5
	Patience	20 epochs
	Threshold	0.0001
	Threshold Mode	‘rel’
	Cooldown	0
	Min Learning Rate	0
	Epsilon	1e-08
Training Strategy	Batch Processing	Mini-batches
	Gradient Calculation	Backpropagation
	Weight Update	Per mini-batch
	Model Checkpointing	Save the best validation loss.
	Sanity Checks	Initial data pipeline and model verification
Performance Metric	Dice Coefficient Calculation	Per batch
Early Stopping	Integrated with Scheduler	Learning rate reduction upon plateauing validation loss
Segmentation Output	Mask Resolution	High-scale
	Upsampling Process	Applied during the decoding phase

### Evaluation measures

For evaluation, the best-performing model comparison was performed in terms of accuracy, precision, recall, F1 score, and Dice coefficient.

#### Accuracy

This calculates the ratio of well-predicted instances to the total number of instances of the model to judge how correct the model is as a whole. This is a primitive and understandable measure, but can be problematic in application, particularly with metrics such as datasets having imbalanced classes. Therefore, it must be accompanied by other measures. The accuracy reflects the overall correctness of the segmentation model based on the proportion of correctly predicted pixels to total pixels in the dataset. Although intuitive and hence easily understandable, accuracy can be very misleading when dealing with datasets containing imbalanced classes. In medical

$$Accuracy = \frac{{TP + TN}}{{TP + TN + FP + FN}}$$

imagery, for example, the head of the fetus would occupy very little space within an image. For this reason, accuracy should be complemented with more specific metrics able to provide a nuanced performance analysis.

Where TP represents true positives, TN represents true negatives, FP represents false positives, and FN represents false negatives.

#### Precision

This represents the number of true positives that are found, considering all the positive predictions the model made. This is an important metric when the cost of false positives—wrongly predicting a positive diagnosis–is extremely high or when it even carries a certain risk. For example, a treated patient diagnosed with cancer, through tests proving positive for the condition, turns out to be free from it. Precision quantifies the fraction of true positives predicted, that is, actual positive pixels to the total positive pixels. Commonly, this is the main measure for

$$\Pr ecision = \frac{{TP}}{{TP + FP}}$$

practical situations where a single false positive carries a relatively high cost; one does not want to perform much over-segmentation, considering only the inside head region. This avoids cases of incorrectly enlarged or irregular shape identification by ensuring the quality of segmentation.

#### Recall (Sensitivity)

The number of positive cases that were predicted correctly. It becomes necessary in cases where the cost or danger of a false negative is high, for example, in screening patients for diseases where a case that has gone undetected may be very serious. Recall or sensitivity is the ability of the model to detect all actual positive pixels. It is calculated as the ratio of true positive pixels to all actual positives in the ground truth mask. High recall is

$$\operatorname{Re} call = \frac{{TP}}{{TP + FN}}$$

necessary for fetal head abnormalities such as microcephaly or macrocephaly, since missing large regions may lead to critical diagnostic errors.

#### F1 score

The F1 score is the weighted average of precision and recall. It can be useful when performance needs to be measured on tasks where precision and recall are usually opposite. This measure is good in situations where neither false negatives nor false positives can be discarded.

$$F1 = 2 \times \frac{{\Pr ecision \times \operatorname{Re} call}}{{\Pr ecision + \operatorname{Re} cal}}$$

The F1 score is a balanced measure because it combines precision and recall through their harmonic mean. In general, it is an important metric when there can be some tradeoff between precision and recall, and it provides full insight into how the model will segment. For example, it ensures that the model correctly identifies the true positive regions while limiting false negatives.

#### Dice coefficient

The Dice Coefficient, sometimes referred to as the Sørensen–Dice Index, is a metric common in medical image segmentation that measures the overlap between predicted and actual segmentation masks. Its values range from 0 (no overlap) to 1 (perfect overlap). The Dice coefficient is thus an indicator in our study of the model’s accuracy of detection and segmentation of the head, which is highly important for small or irregular head shapes, such as those associated with microcephaly or macrocephaly, during the measurement of head circumference and the biparietal diameter. That is, the Dice coefficient needs to be weighed against other metrics because it provides a direct measure of model precision in head identification.

$$\:Dice\:Coefficient = \frac{{2 \times \:\left| {A \cap B} \right|}}{{\left| A \right| + \left| B \right|}}$$

Where A is the set of predicted pixels and B is the set of ground truth pixels.

Finally, the best model is compared with 200 ultrasound images that are annotated, measured, and classified by industry expert radiologists. These metrics help in presenting the effectiveness of the model in a balanced way, i.e., taking into consideration the true positive predictions as well as the costs of misclassifications incurred.

## Results

### Proposed model performance

Under the same environment as those of the proposed five models, an experiment was performed to choose the most promising model for further optimization.

**Table 5:** Proposed model performances in the same environment

Models	SegNet	UNet	FCN	MobileNetV2	EfficientNet-B0
Accuracy	0.7173	0.7113	0.6842	0.9652	0.9650
Precision	0.7798	0.8075	0.0001	0.9804	0.9636
Recall	0.1461	0.1129	0.0001	0.9079	0.9240
F1 Score	0.2461	0.1982	0.0001	0.9428	0.9434
Dice Coefficient	0.3270	0.2738	0.0001	0.0199	0.01316

**Fig. 4:**
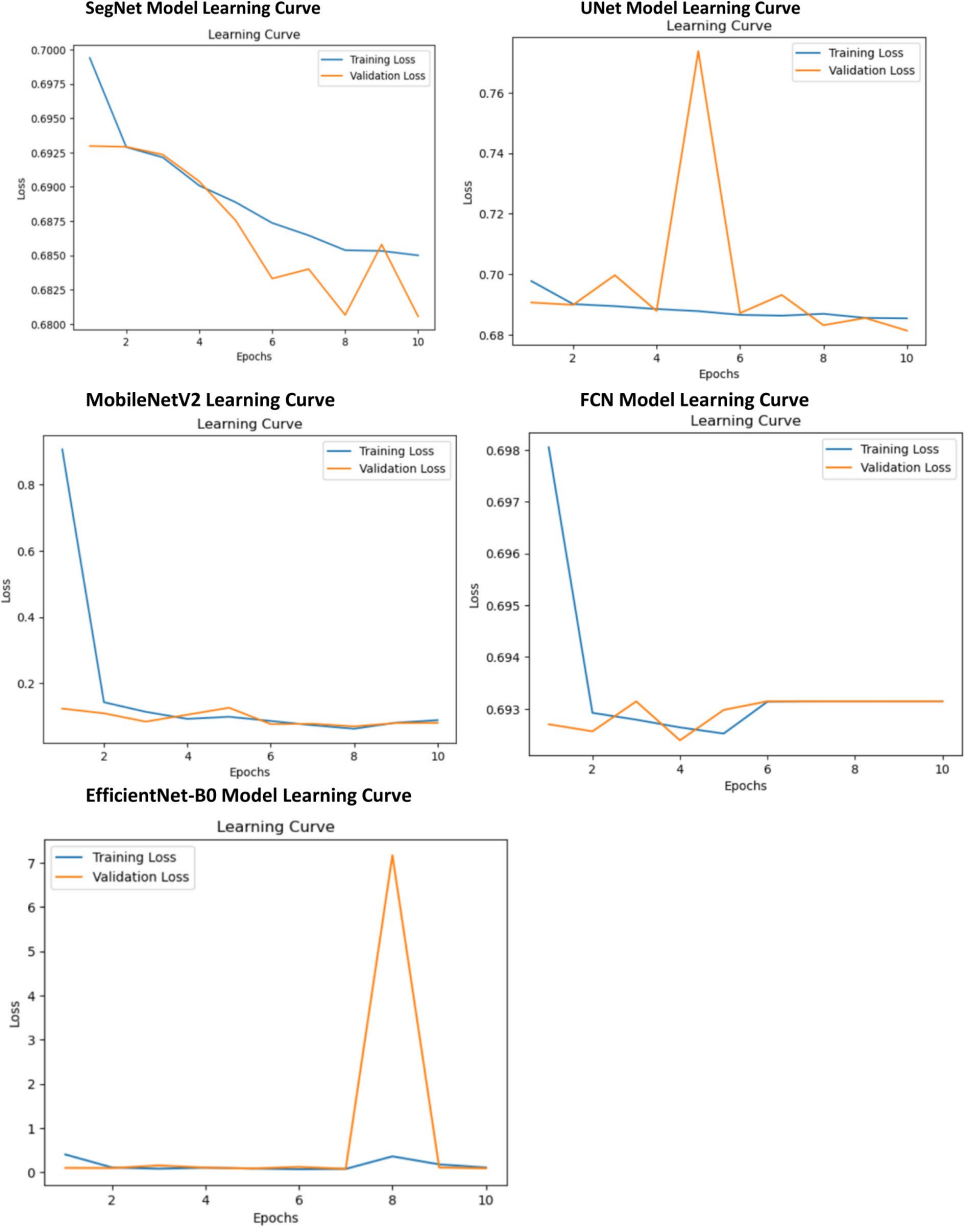
First experimental results

Among all the models, SegNet achieves the highest Dice coefficient of 0.3270, indicating its superior segmentation performance. It also recorded moderate accuracy (0.7173) and precision (0.7798). The model’s consistent decrease in training and validation loss highlights its effective learning and generalization capabilities, making it a promising choice for segmentation tasks. U-Net ranked as the second-best model, achieving a Dice coefficient of 0.2738 and an accuracy of 0.7113 (Table 5). While slightly behind SegNet, it demonstrated respectable precision and remains a strong candidate for further development. The FCN demonstrated poor performance, with a Dice coefficient of 0.0001, indicating an inability to learn meaningful features during training and rendering it unsuitable for the segmentation task in its current configuration. MobileNetV2 and EfficientNet-B0 achieved high accuracy scores of 0.9652 and 0.9650, respectively, alongside strong precision. However, both exhibited low Dice coefficients (0.0199 for MobileNetV2 and 0.01316 for EfficientNet-B0), highlighting significant issues with segmentation mask overlap. EfficientNet-B0 also showed erratic learning behavior, including a spike in validation loss, suggesting overfitting or instability [Fig. 4]. These results underscore their limitations for segmentation tasks. This indicates that SegNet is the top-performing model and may be further optimized with hyperparameter tuning, sophisticated data augmentation, and custom loss functions. UNet has more potential for modifying its architecture. The FCN requires major adjustments because it fails to learn properly [Fig. 4]. MobileNetV2 and EfficientNet-B0 resulted in high accuracy, while their low Dice coefficients underlined some critical limitations related to segmentation, which highlights the importance of choosing metrics and further evaluation.

### Model optimization results

The optimization of the UNet model led to significant improvements in its performance, as depicted in the learning curves. The training and validation losses demonstrated a steady decline over the 50 epochs, with both curves converging smoothly. This indicates effective learning and reduced overfitting. The training loss decreased from an initial value of approximately 0.58 to approximately 0.51, whereas the validation loss exhibited a similar trend, starting at approximately 0.55 and stabilizing at approximately 0.49.

**Fig. 5:**
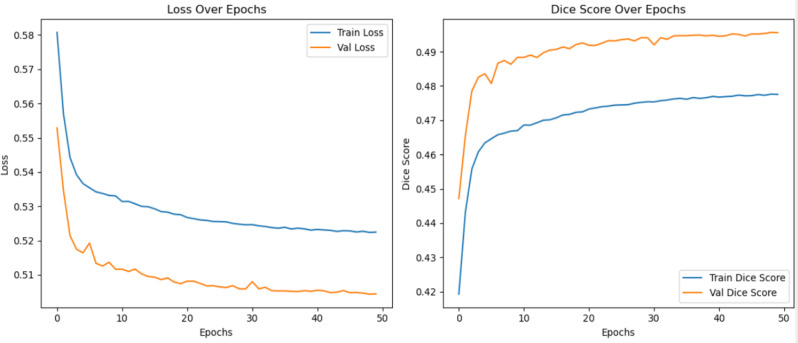
UNet model performance after optimization

The Dice score, a crucial metric for segmentation performance, also shows a marked improvement. The training Dice score increased consistently from an initial value of 0.42 to approximately 0.48 by the end of training, whereas the validation Dice score rose from 0.45 to 0.49, reflecting robust model generalizability. While showing improvement in terms of loss and Dice score, there are signs of underfitting [Fig. 5].

**Fig. 6:**
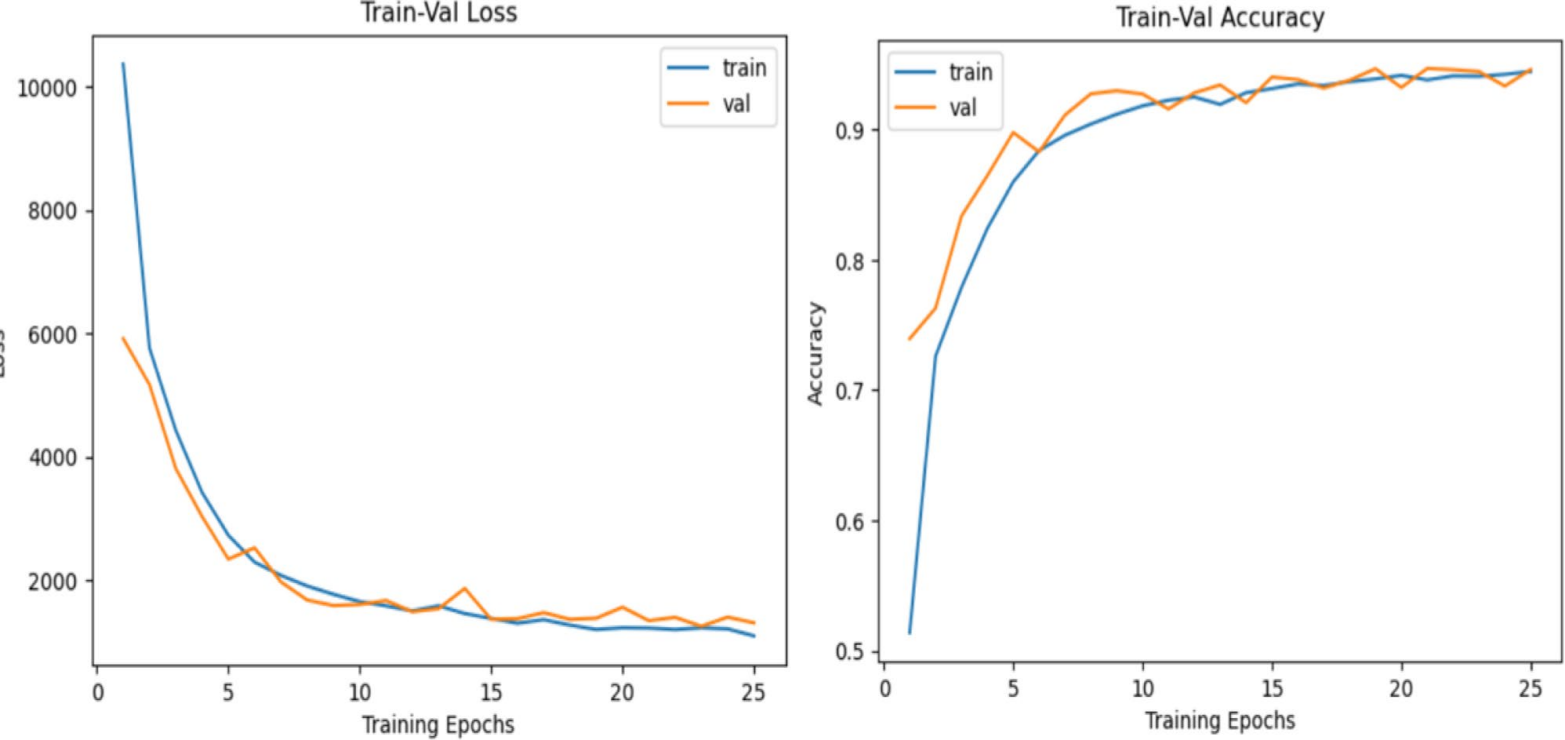
Optimized SegNet performance at 25 Epochs

The optimization of the SegNet model resulted in a remarkable improvement in both training and validation performance, as evidenced by the learning curves over 25 epochs. The training and validation loss curves significantly decreased, with the training loss decreasing steeply from an initial value of over 10,000 to approximately 1,500, whereas the validation loss followed a similar trend, stabilizing near the same level (Fig. 6). The proximity of the training and validation loss curves highlights the model’s ability to generalize well without overfitting.

The accuracy metrics further reinforce SegNet’s strong performance. The training accuracy improved rapidly from an initial value of approximately 50% to exceeding 90%, whereas the validation accuracy closely tracked this progress, stabilizing at a similarly high value. This demonstrates the model’s ability to effectively learn and generalize across the dataset, owing to the incorporation of advanced data augmentation techniques and architectural adjustments.

**Fig. 7:**
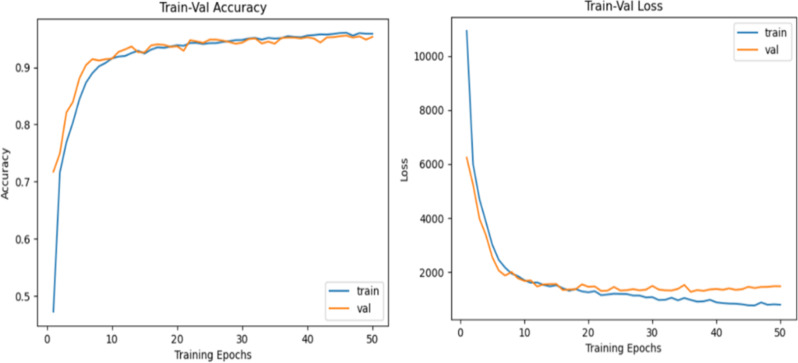
Performance of the SegNet model after tuning at 50 epochs

After training the SegNet model for 50 epochs, the performance metrics indicated further refinement in the model’s learning capabilities. The training and validation loss curves consistently decreased, stabilizing at approximately 1,000 by the end of the training process. This stability in both curves reflects strong generalization to unseen data, with no significant indications of overfitting, even with extended training. The accuracy metrics also demonstrated robust performance, with the training accuracy exceeding 95% and the validation accuracy closely following, stabilizing near the same range (Fig. 7). This consistency between training and validation accuracy highlights that the model has effectively captured the underlying data patterns while maintaining generalizability.

Compared with training for 25 epochs, the additional epochs provided further fine-tuning of the model, leading to marginally improved accuracy and reduced loss. This demonstrates the effectiveness of prolonged training for SegNet, enabling it to extract more refined features from the dataset. These results underscore SegNet’s ability and robustness for segmentation tasks, especially when it is provided with extended training iterations. The outcomes further validate its suitability for applications that require precise and reliable segmentation performance.

After training the SegNet model for 100 epochs, the performance metrics indicate further fine-tuning and stability in the model’s behavior. The training and validation loss curves show a consistent downward trend, with the training loss decreasing to approximately 500 and the validation loss stabilizing around a similar value. The fluctuations in the validation loss curve observed in the latter epochs reflect minor variability, possibly due to slight changes in the validation dataset or the optimization process, but they do not indicate overfitting.

The training and validation accuracy curves depict exceptional performance, both exceeding 95% and approaching 98% by the final epoch. This high level of accuracy underscores the model’s effectiveness in learning the complex features of the dataset. Importantly, the close alignment of training and validation accuracy indicates that the model has achieved strong generalizability and has avoided overfitting, even with extended training.

The extension of the training period to 100 epochs allows the model to further refine its understanding of the data, capturing intricate patterns and details. However, the minimal improvement in performance metrics beyond 50 epochs suggests that the model has likely reached its peak learning capacity. At this point, further training might yield diminishing returns and risk overfitting or unnecessary computational expenses.

**Fig. 8:**
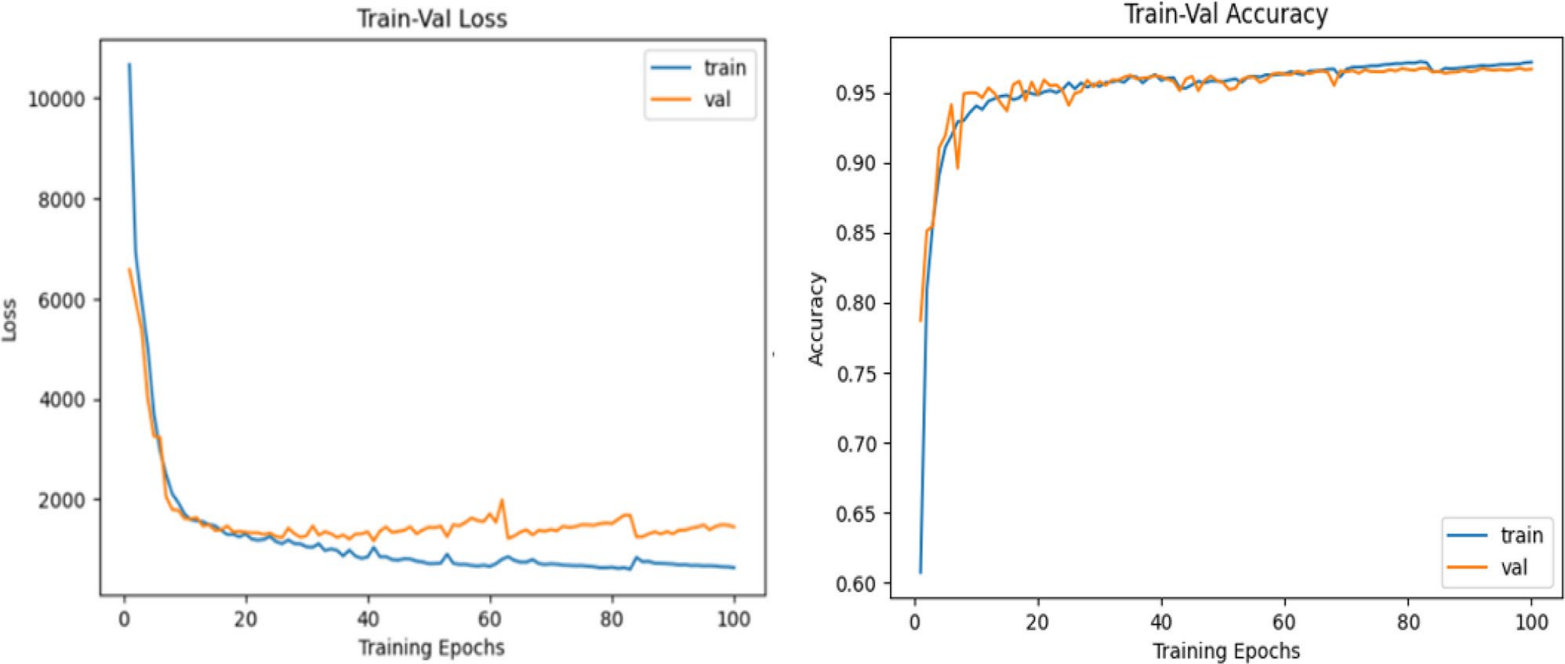
Performance of the SegNet model after tuning for 100 epochs

In conclusion, training SegNet for 100 epochs results in a highly effective segmentation model with excellent accuracy and low loss values, with an accuracy of 98% and a Dice coefficient of 0.97 (Fig. 8). This demonstrates its robustness and reliability for real-world segmentation tasks, particularly in scenarios requiring high precision.

Training the SegNet model beyond 100 epochs was found to be inefficient when it was experimented with 125 epochs, as the model demonstrated signs of overfitting and diminishing returns in performance. While the initial increase in epochs allowed the model to refine its learning and achieve better segmentation accuracy, further training beyond 100 epochs did not result in meaningful improvements. The Dice coefficient, a critical metric for evaluating segmentation overlap, starts to stagnate and even slightly diminishes, indicating that the model has reached its learning capacity within this dataset. Additionally, the validation loss begins to plateau and exhibits minor fluctuations, further suggesting overfitting. These patterns confirm that the model had effectively learned the task by the 100th epoch, and additional training only risked degrading its generalization performance. Based on these observations, 100 epochs were chosen as the optimal training duration for balancing learning efficiency and performance while minimizing the risk of overfitting. This decision ensures that the model achieves its best possible performance without unnecessary computational overhead or degradation in its predictive accuracy.

### Segmentation results from the model

**Fig. 9:**
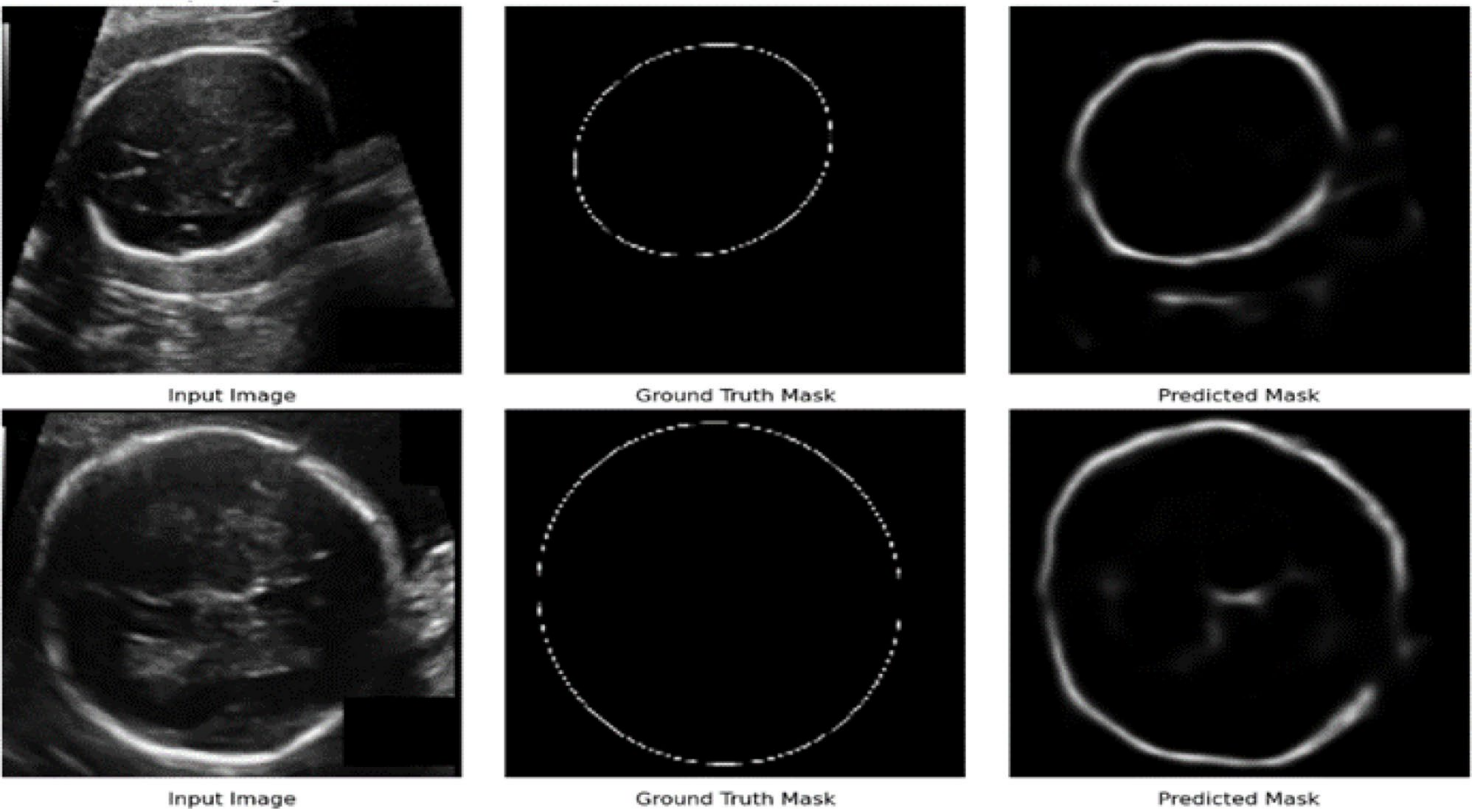
Segmentation result of optimized segNet the model

The SegNet model demonstrated promising performance in segmenting fetal head boundaries from ultrasound images, closely approximating ground truth masks. The predicted masks effectively captured the overall head structure, and slight edge blur and inconsistencies were noted due to ultrasound image noise and variations [Fig. 9].

**Fig. 10:**
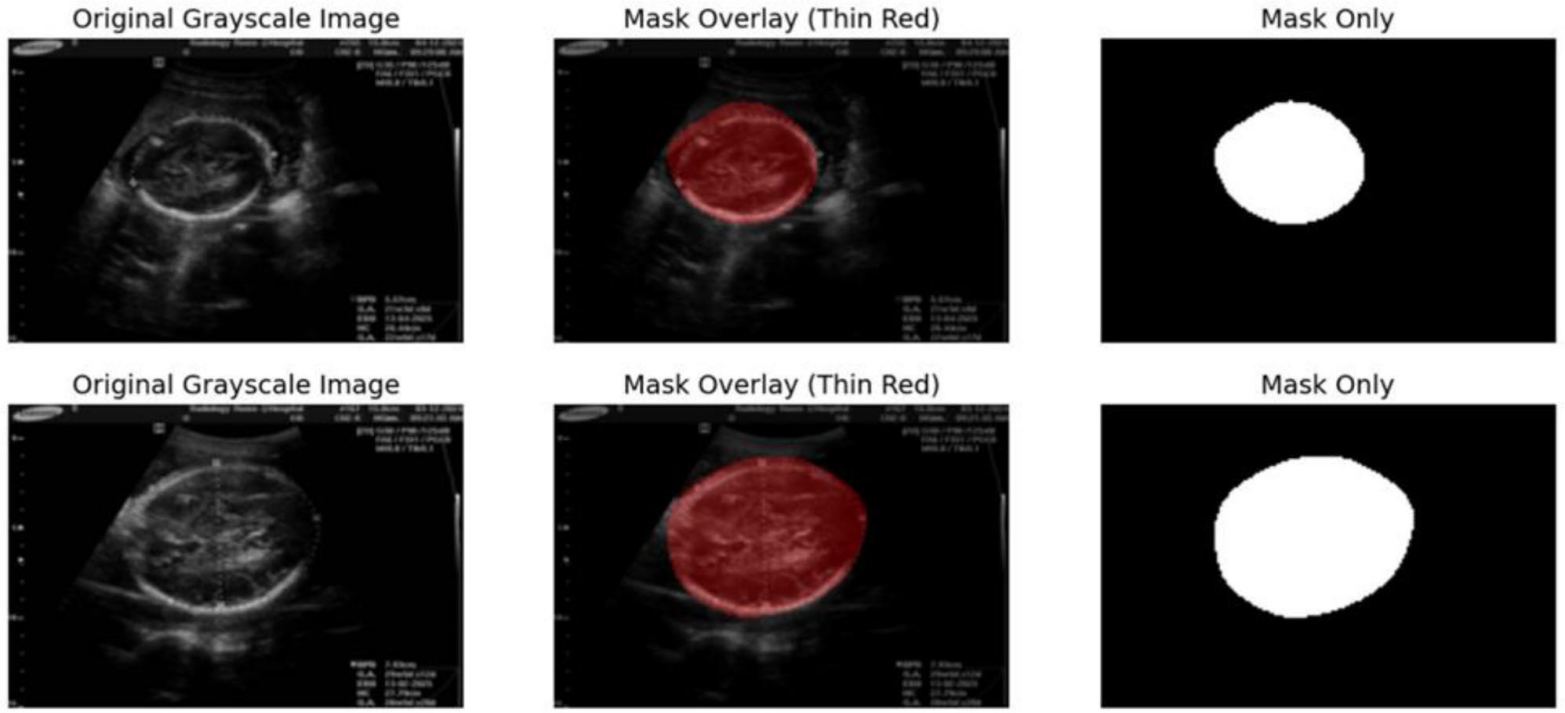
Segmentation result of the model with predicted mask and ground truth

### Measurement of HC and BPD

Segmentation masks generated from the SegNet model are used for extracting biometric measurements, which include HCs and BPDs. These clinically important measurements are derived directly from the predicted masks to illustrate the strengths of the model for effective automated quantitative analysis of the fetal head. The clear boundaries of the masks highlight the strength of the SegNet model in segmenting the head region accurately, which is very important in identifying abnormalities such as microcephaly and macrocephaly.

Segmentation of the fetal head via the SegNet model. The output of segmentation is presented in three formats: the original grayscale image, the mask overlay, and the mask only. The Original Grayscale Image represents the fetal head as captured in the ultrasound scan. Mask Overlay (Thin Red: In the grayscale image, the segmentation mask generated by the SegNet model is superimposed, highlighting the exact boundary of the fetal head. Mask Only: This column isolates the segmentation result where the head region is represented as white against a black background [Fig. 10].

**Fig. 11:**
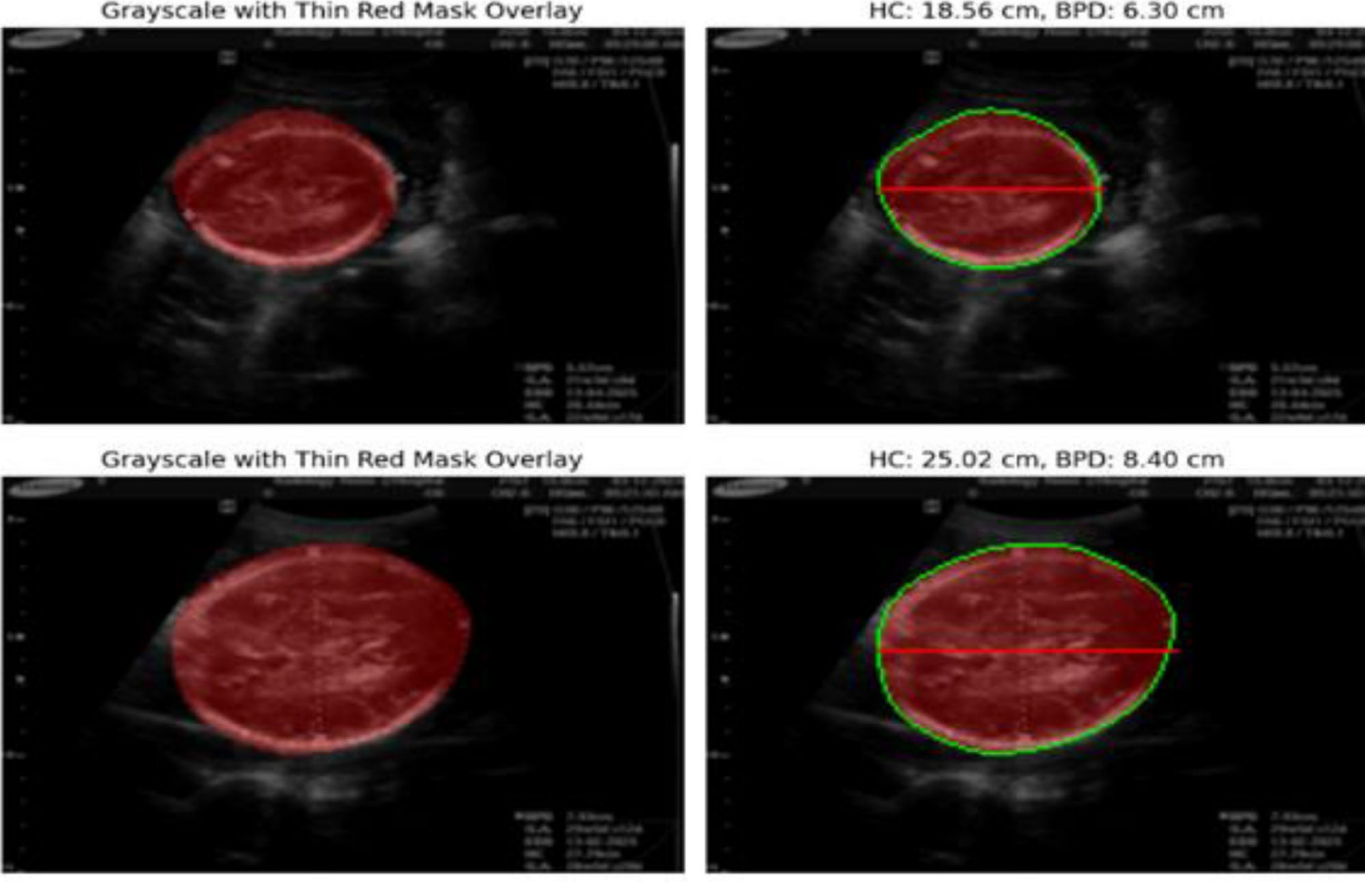
Measurement of HC and BPD

SegNet performs the prediction of segmentation masks from which biometric measurements, such as HC and BPD, are extracted. Such clinical measurement estimates that directly result from these predicted masks illustrate the strengths of this model for automated, quantitative analysis of the fetal head. Moreover, the sharp contrast of boundaries in the masks shows that the SegNet model has high performance in segmenting the head region quite precisely, which is a critical factor for identifying abnormalities such as microcephaly and macrocephaly [Fig. 11].

### Model performance in comparison with industry experts

In addition to measurement accuracy, our framework involves a classification module for categorizing fetuses into macrocephalic, microcephalic, and normocephalic classes based on the measured values of HCs. Macrocephaly was clinically defined as an HC above the 98th percentile or greater than + 2 SDs above the mean for gestational age. Conversely, microcephaly is defined as an HC more than 2 SDs below the average for age and sex. HC measurements that fall within normal limits for gestational age are considered normal.

On this basis, a comparison with industry experts is made. These results show that our model can obtain a specificity of 89.3%, a sensitivity of 93.5%, and an accuracy of 91.2% in the case of BPD measurement, whereas, in the case of HC measurement, the model attained a specificity of 85.0%, a sensitivity of 94.2%, and an accuracy of 92.5%. These metrics confirm that our approach is robust and reliable, providing both BPD and HC with close approximations to expert-level accuracy [Table 6].

**Table 6:** Performance of the model compared with that of an expert

Metric	Specificity (%)	Sensitivity (%)	Accuracy (%)
BPD	89.3	93.5	91.2
HC	85.0	94.2	92.5

### Model prototype

The web application developed for this research will support medical professionals in the segmentation of fetal heads and the measurement of important parameters, such as HCs and BPDs, from ultrasound images. This tool uses advanced deep-learning techniques to obtain accurate and reliable measurements, which are critical for assessing fetal growth and development.

**Fig. 12:**
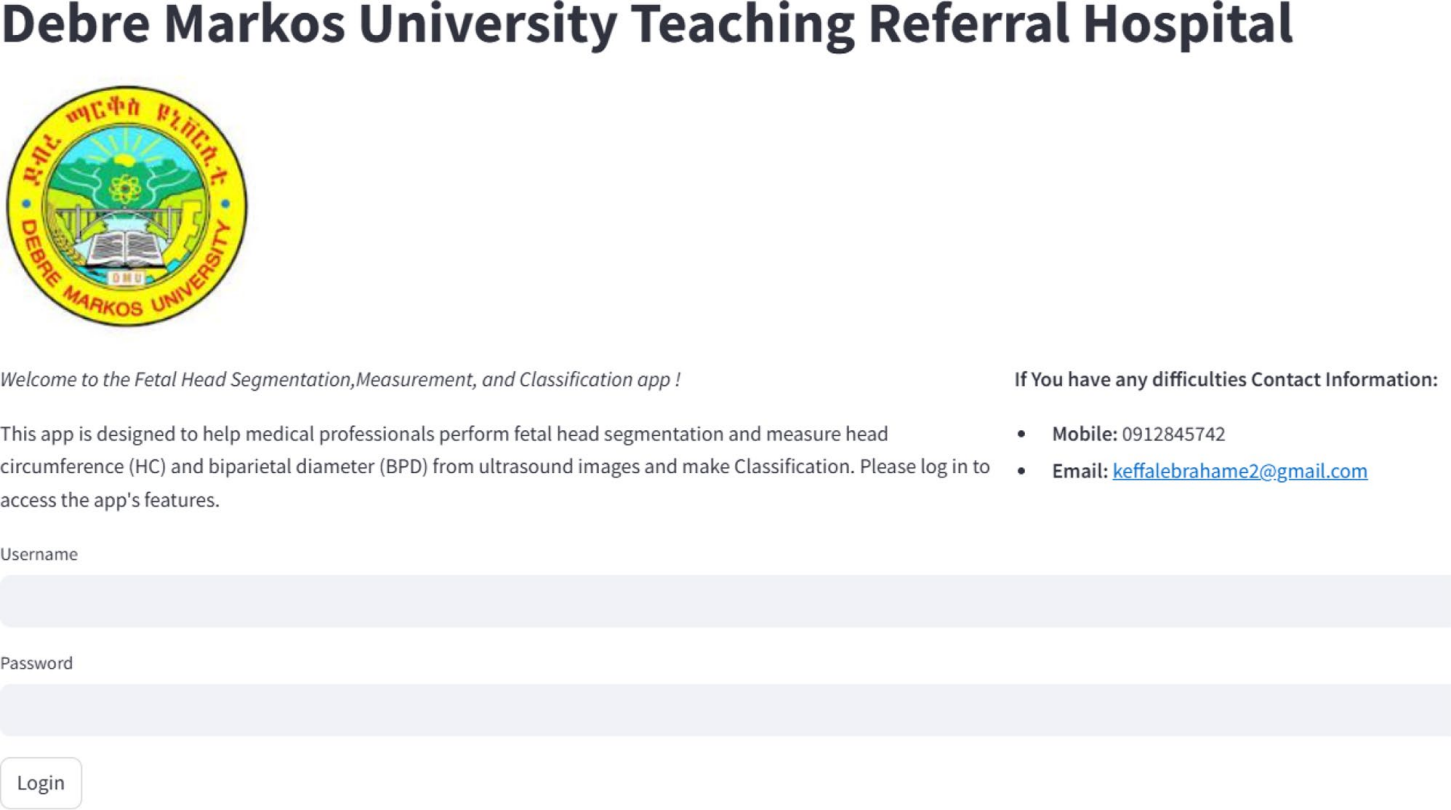
Model prototype output

The application is developed on Streamlit, a high-end Python framework that helps in crafting interactive web applications. Everything has been intuitively designed so that all functions are very user-friendly for medical professionals. When the application opens, the user views a login screen for secure access. From this page, the application presents important details, including but not limited to, the purpose of the application, what to expect from the user, and how one can get in touch for support. Upon successful login, users can upload ultrasound images of the fetal head. After that, this application preprocesses these images through the Albumentations library, which involves resizing and all the necessary transformations of the images. At the center of this application is a deep learning model that performs accurate segmentation of the fetal head within ultrasound images via the SegNet architecture [Fig. 12]. The model used in this system is implemented via PyTorch.

It overlays the segmented mask onto the original image to present a visual result regarding segmentation. Then, contour analysis and region properties are used to calculate the HC and BPD via the program. The results of the measurements are presented to the user, along with the expected ranges in cases of different gestational ages. These measurements are then classified by the application into categories such as “normal,” “microcephaly,” or “macrocephaly” based on predefined rules.

**Fig. 13:**
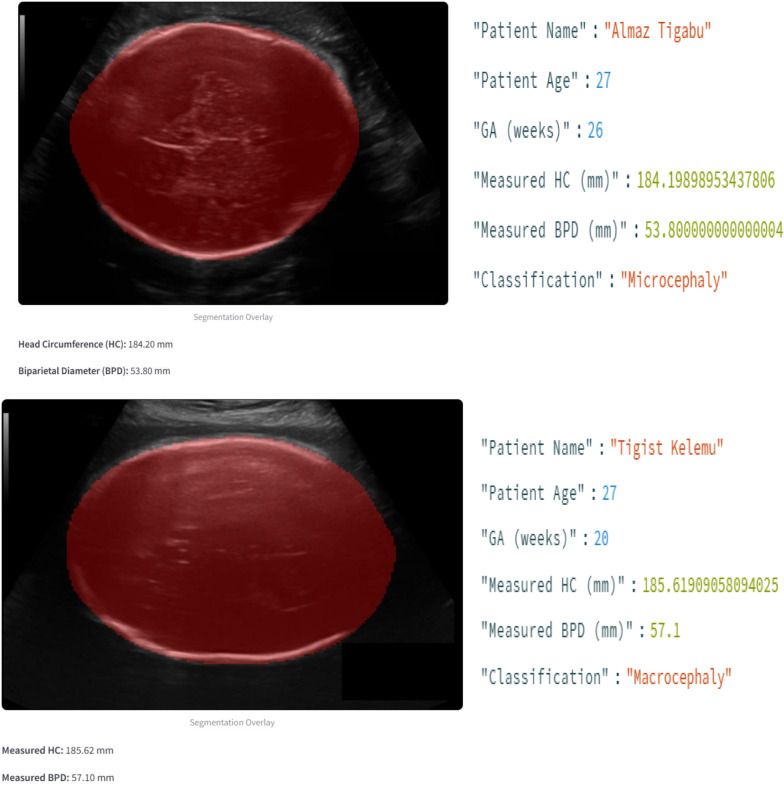
Model prototype output

The application allows the user to input the name and age of the patient to enable detailed reporting. These, along with the measurements and classification, are combined into a detailed report that can be downloaded as a PDF showing the original ultrasound image and all the information. This is very useful for record-keeping purposes and sharing results with other healthcare professionals. The application also includes a logout functionality, ensuring that user sessions are securely managed (Fig. 13).

Overall, this web application represents a significant advancement in the field of fetal health assessment, providing a reliable and efficient tool for medical professionals.

## Discussion

This work illustrates the high performance of SegNet in segmenting ultrasound images of the fetal head to solve some traditional and modern challenges. Ultrasound imaging is intrinsically problematic because of low contrast, noise, and variability in the shape of fetal heads. Previous related works using conventional image processing techniques, such as edge detection and region-growing algorithms, often faced difficulties with such problems. This is because previous works [20, 21] have reported that these approaches normally fail in instances of irregular head boundaries or low-quality images. SegNet showed strong segmentation that could present head boundaries from different imaging conditions.

SegNet also outperforms other recent deep learning methods. For example, UNet is among the most commonly used architectures for medical image segmentation tasks [22–24]. However, there are well-known limitations in maintaining spatial information during the up-sampling process. These limitations affected its performance in this work, with a Dice coefficient of 0.2738 and an accuracy of 0.7113. Similar findings have been reported in prior studies, where UNet struggled in tasks requiring precise boundary delineation because it relied on simple up-sampling mechanisms [25].

Another well-known deep learning approach, fully convolutional networks, performs very poorly on this task and fails to converge effectively. This is further demonstrated by its flat learning curves and Dice coefficient of 0.0001; hence, it is unable to handle the complexity of fetal head segmentation in ultrasound images. This result agrees with earlier works where the inability of FCNs to preserve spatial details was limiting in medical imaging tasks [26]. In contrast, SegNet uses pooling indices to preserve crucial spatial information during up-sampling and achieves an initial Dice coefficient of 0.3270 and an accuracy of 0.7173. These results emphasize how architectural design plays a significant role in achieving superior segmentation performance.

With systematic optimization, including hyperparameter tuning, advanced data augmentation, and refined loss functions, SegNet achieved an overall accuracy of 98%, considerably outperforming both traditional approaches and other deep learning models. Previous related works using deep learning for fetal head segmentation reported accuracies within the range of 85-92% [27–29]. The fact that SegNet outperforms these benchmarks underlines its robustness and adaptability to challenging conditions.

The performance of SegNet for automated biometric measurements of HCs and BPD patients was also quite impressive. The model attained an accuracy of 93% for both measurements, with sensitivity and specificity closely matching expert annotations. Most previous studies were based on manual or semiautomated measurement techniques, which, although accurate, are quite time-consuming and prone to interobserver variability [30, 31]. For example, one semiautomated study reported an average accuracy of 89% for HC measurements but noted significant variability among observers [32]. The improved accuracy and reduced variability offered by SegNet represent a significant advance in the field.

In addition to achieving high accuracy, SegNet al.so shows consistency in challenging situations, such as irregular fetal head shapes and noisy conditions. Previous research has more often reported difficulties in such challenging situations, where segmentation errors vary between 10% and 20% depending on the task’s difficulty [33, 34]. The fact that SegNet was able to ensure high accuracy in these cases allows it to be highlighted for daily clinical usage.

One of the key strengths of this work is its use of both HC (HC) and BPD (BPD) for classifying macrocephaly and microcephaly. This approach demonstrates improved accuracy compared with earlier studies that relied solely on HC measurements [35].

Traditional approaches for fetal head segmentation are based on edge detection or region-growing algorithms. However, these low-level image features often result in poor performance for images with noise or low contrast. Accuracies for conventional methods have been reported within the range of 70–80%, and such methods are likely to exhibit significant variability under challenging conditions [33]. In contrast, SegNet achieved 98% accuracy, showing the ability to overcome some of these barriers.

Since the UNet architecture contains an encoder-decoder structure with skip connections, it is among the most famous medical image segmentation models. However, many studies prove that UNet suffers from the loss of spatial information during up-sampling [25]. In this work, UNet had a Dice coefficient of 0.2738 and an accuracy of 0.7113, considerably lower than the initial performance of SegNet. This also agrees with several previous studies where the performance of UNet was bound by its architecture.

FCNs have been applied to a variety of segmentation tasks, but usually face difficulties with complex medical images. Its inability to preserve spatial details during up-sampling was evident in this study, where it failed to converge and achieved a Dice coefficient of 0.0000. Previous studies have reported similar challenges, with FCNs often underperforming in tasks requiring precise boundary delineation [26]. SegNet’s use of pooling indices to retain spatial details addresses this limitation, enabling superior performance.

Recent studies have investigated the use of advanced architectures, including DeepLab and ResNet-based models, for medical image segmentation. While those models demonstrate very promising results, their computational complexity and extensive training generally limit their practical use [36]. SegNet provides a good trade-off between performance and computational efficiency; hence, it is a feasible option for clinical applications.

These results have several important implications for clinical practice and future research. First, the high accuracy and robustness of SegNet in fetal head segmentation could significantly reduce manual interventions and, therefore, smooth clinical workflows, enhancing diagnostic efficiency. The model’s ability to achieve accurate biometric measurements with minimal variability further underlines its potential to improve prenatal care.

Second, the architecture of the deep learning model while performing medical imaging tasks is identified. This is an advance over conventional up-sampling techniques that use pooling indices from SegNet, enabling this network to yield outstanding results even under the worst conditions. On this basis, future deep-learning models will need novel architectural designs.

Third, the study identifies potential systematic optimization, hyperparameter tuning, and data augmentation, which have been proven to provide considerable improvements, although this could be transferable to most medical image tasks as long as accuracy and robustness are desirable.

For the future, the use of more complex models, such as 3D convolutional networks and generative adversarial networks, which may further improve detection performance and handle variability in image quality and fetal positioning, is suggested. These more advanced models, trained on a richer and more diverse dataset, have the potential to outperform current approaches and provide more reliable and scalable solutions for prenatal care across the country.

### Limitations of the study

The primary limitation of this study is the relatively small dataset size, consisting of 700 ultrasound images collected from three healthcare facilities. While efforts were made to ensure a balanced representation across trimesters and facilities, the dataset may not fully capture the diversity of fetal head shapes, imaging conditions, and population characteristics. This limitation could affect the generalizability of the model to broader clinical settings. Future research should focus on expanding the dataset by incorporating images from additional healthcare facilities and different geographic regions to enhance the model’s robustness and applicability. While our model identifies biometric deviations associated with microcephaly and macrocephaly risk, it does not replace clinical diagnosis. Definitive diagnosis requires postnatal confirmation and etiological evaluation, which were beyond the scope of this study.

## Conclusion

The results establish SegNet as the state-of-the-art model for fetal head segmentation, biometric measurements in ultrasound imaging, and classification of macrocephaly and microcephaly. With the solution of the main drawbacks in the traditional and state-of-the-art approaches, SegNet renews the benchmark for accuracy and robustness regarding the topic. The results highlight how deep learning can be useful in prenatal diagnostics by positively impacting the clinical workflow and patient outcomes. With further research and development, SegNet can become a cornerstone of automated diagnostic systems, driving innovations in prenatal care and setting the stage for future advancements in medical imaging.

## Data Availability

The datasets used and/or analyzed during the current study are available from the corresponding author upon reasonable request. The analysis is freely available on our public GitHub repository (https://github.com/abrahamekeffale/Fetal_detection_project.git).

## Abbreviations

AI: Artificial Intelligence
ANN: Artificial Neural Network
API: Application Programming Interface
AUC: Area under the curve
BPD: Biparietal diameter
CMHS: College of Medicine and Health Sciences
CNN: Convolutional Neural Network
CPU: Central Processing Unit
ETB: Ethiopian Birr
FCN: Fully Convolutional Network
GA: Gestational Age
GAN: Generative adversarial network
GCP: Good clinical practice
GPU: Graphics Processing Unit
HC: Head Circumference
ICH: International Council for Harmonization
IDE: Integrated development environment
IORG: Institutional Official Registration for Human Research Protections Program
IRERC: Institutional Review and Ethics Review Committee
ML: Machine Learning
MRI: Magnetic Resonance Imaging
RAM: Random Access Memory
ROC: Receiver operating characteristic
XAI: Explainable AI
